# Fetal cardiac parameters for predicting postnatal operation type of fetuses with tetralogy of Fallot

**DOI:** 10.1186/s12947-022-00274-5

**Published:** 2022-02-21

**Authors:** Suyeon Park, Hye-Sung Won, Rina Kim, Mijin Kim, Jeong Jin Yu, Chun Soo Park, Tae-Jin Yun, Yewon Jung, Usamah Al Harbi, Mi-Young Lee

**Affiliations:** 1 Department of Obstetrics and Gynecology, University of Hallym College of Medicine, Hallym Sacred Heart Hospital, Anyang, South Korea; 2 Department of Obstetrics and Gynecology, University of Ulsan College of Medicine, Asan Medical Center, Seoul, South Korea; 3 Department of Obstetrics and Gynecology, Jeju National University College of Medicine, Jeju National University Hospital, Jeju, South Korea; 4 Department of Pediatrics, Division of Pediatric Cardiology, University of Ulsan College of Medicine, Asan Medical Center Children’s Hospital, Seoul, South Korea; 5 Division of Pediatric Cardiac Surgery, University of Ulsan College of Medicine, Asan Medical Center, Seoul, South Korea; 6 Department of Obstetrics and Gynecology, Chungnam National University College of Medicine, Chungnam National University Sejong Hospital, Sejong, South Korea

**Keywords:** Fetal echocardiography, Pulmonary artery, Tetralogy of Fallot

## Abstract

**Background:**

To assess fetal cardiac parameters predictive of postnatal operation type in fetuses with tetralogy of Fallot (TOF).

**Methods:**

Echocardiographic data obtained in the second and third trimesters were retrospectively reviewed for fetuses diagnosed with TOF between 2014 and 2018 at Asan Medical Center. The following fetal cardiac parameters were analyzed: 1) pulmonary valve annulus (PVA) z-score, 2) right pulmonary artery (RPA) z-score, 3) aortic valve annulus (AVA) z-score, 4) pulmonary valve peak systolic velocity (PV-PSV), 5) PVA/AVA ratio, and 6) RPA/descending aorta (DAo) ratio. These cardiac parameters were compared between a primary corrective surgery group and a palliative shunt operation followed by complete repair group.

**Results:**

A total of 100 fetuses with TOF were included. Only one neonatal death occurred. Ninety patients underwent primary corrective surgery and 10 neonates underwent a multistage surgery. The PVA z-score, RPA z-score, and RPA/DAo ratio measured in the second trimester and the PVA z-score, RPA z-score, and PVA/AVA raio measured in the third trimester were significantly lower in the multistage surgery group, while the PV-PSV as measured in both trimesters were significantly higher in the multistage surgery group.

**Conclusion:**

Fetal cardiac parameters are useful for predicting the operation type necessary for neonates with TOF.

## Background

Tetralogy of Fallot (TOF) is the most common form of cyanotic heart disease, with an incidence of one per 3600 live births and affecting 5 to 7% of infants with congenital heart diseases [1] [2, 3]. The diagnosis of TOF is commonly made during the fetal period with a high degree of accuracy [1, 3]. TOF is described by four classic anatomical characteristics: outlet ventricular septal defect (VSD) with anterior malalignment, overriding aorta, pulmonary stenosis (PS), and right ventricular hypertrophy. According to the degree of right ventricular outflow tract (RVOT) obstruction, TOF can manifest as a variety of clinical presentations ranging from asymptomatic detection to early postnatal cyanosis requiring urgent treatment. Ultimately, TOF with the exception pf pulmonary atresia with VSD can be divided into two types depending on the size of the pulmonary artery (PA) and the type of surgery. Type 1 patients, who have normal-sized PA and mild PS, usually undergo primary corrective surgery in the first year of life [4, 5] [6]. However, type 2 patients, who have small-sized PA and severe cyanosis, require multistage surgery consisting of, first, a palliative shunt operation that is subsequently followed by complete repair in later months [4–6].

In the fetal period, information on predictive factors to determine the operation type necessary in neonates with TOF is limited. In this study, we evaluated the cardiac parameters of fetuses prenatally diagnosed and postnatally confirmed to have TOF to support the prediction of postnatal operation type.

## Materials and methods

This was a retrospective cohort study of fetuses prenatally diagnosed and postnatally confirmed to have TOF at Asan Medical Center, Seoul, Korea between January 2014 and December 2018. The study protocol was approved by the institutional review board of Asan Medical Center (approval no. 2020–0463). Formal consent was not required because it was a retrospective study. All prenatal ultrasonographic examinations were performed using a WS80A (Samsung Medison Co., Ltd., Seoul, Korea) or a Voluson E8 or E10 Expert (GE Healthcare Austria GmbH & Co. OG, Zipf, Austria) with a 2- to 6-MHz transabdominal probe. The inclusion criteria were patients with simple TOF who were followed up longitudinally with at least twice by echocardiographic evaluations in the second and third trimesters, respectively, and who were born at our center. Patients with combined minor cardiac anomalies, such as right aortic arch, aberrant right or left subclavian artery, or persistent left superior vena cava, were also included. Meanwhile, patients with pulmonary atresia with VSD, an absence of pulmonary valve syndrome, or other associated complex cardiac anomalies were excluded.

Relevant prenatal and postnatal data collected for this study included the maternal age; gestaional age (GA) at diagnosis, follow-up, and delivery; rate of preterm birth; birth weight; Apgar score at 1 minute and 5 minutes; associated chromosomal and extracardiac anomalies; and occurence of neonatal death.

**Fig. 1:**
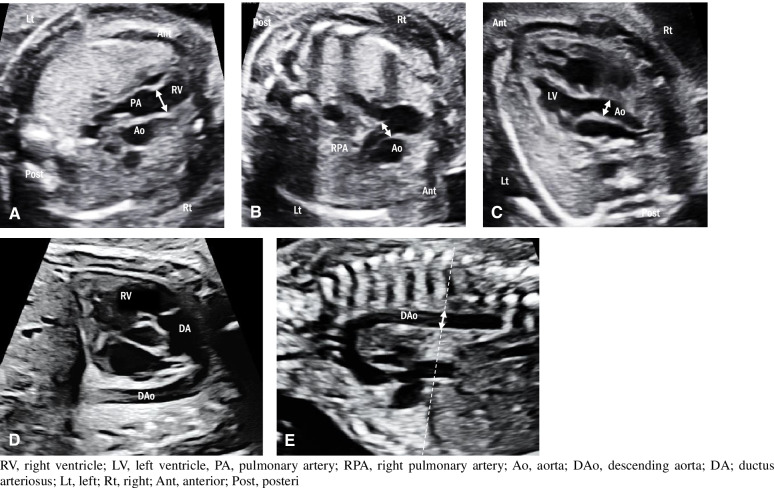
Measurements of the fetal cardiac parameters. In the right ventricular outflow tract view, pulmonary valve annulus is measured from hinge point to hinge point at the location of maximal expansion during peak systole (**A**, arrow). In the three-vessel view, the right pulmonary artery is measured at the proximal part of its bifurcation (**B**, arrow). In the left ventricular outflow tract view, the aortic valve annulus is measured from hinge point to hinge point at the location of maximal expansion during peak systole (**C**, arrow). In the ductal arch view, pulmonary valve peak systolic velocity is measured at the level of the pulmonary valve using pulsed Doppler (**D**). In the aortic arch view, the descending aorta is measured at the level of the diaphragm (E, arrow)

Fetal echocardiography was conducted in all cases. The following fetal cardiac parameters were analyzed: 1) pulmonary valve annulus (PVA) z-score, 2) right PA (RPA) z-score, 3) aortic valve annulus (AVA) z-score, 4) pulmonary valve peak systolic velocity (PV-PSV), 5) PVA/AVA ratio, and 6) RPA/descending aorta (DAo) ratio (modified McGoon ratio). Valve annuli were measured from hinge point to hinge point at the location of maximal expansion in the long-axis view of the heart during peak systole (Fig. 1A and C) [7, 8]. The RPA was measured in either the three-vessel view or oblique short-axis view, yielding a longitudinal view of the PA and its bifurcation (Fig. 1B). Pulsed Doppler was adopted to assess the PV-PSV in the ductal arch view or right long-axis view (Fig. 1A and D). Doppler velocity was recorded with the angle of insonation not exceeding 15° and with at least five uniform waveforms required for the measurements. The DAo was measured in the aortic arch view at the level of the diaphragm (Fig. 1E). Z-scores were calculated using normative data adjusted to GA [7]. During the study period, several experts in fetal echocardiography performed examinations using the same methodology. A single investigator (S. P.) retrospectively re-measured the following fetal cardiac parameters twice using the stored images and the mean values of such were used for the analysis. The Cronbach’s alpha reliability coefficient was used to evaluate intra-observer variability and the result was 0.981, which showed excellent reliability.

All patients underwent either single-stage (primary corrective surgery) or multistage surgery (palliative shunt operation followed by complete repair). The choice of operation was made by pediatric cardiologists and pediatric cardiac surgeons, depending on the severity of the RVOT obstruction, including clinical symptoms (e.g., cyanosis and spell events) and the McGoon ratio and the Nakata index as measured by postnatal transthoracic echocardiography or cardiac computed tomography (CT). The McGoon ratio was calculated as the sum of diameter of the left PA (LPA) and RPA at the prebranching point divided by the diameter of the DAo at the level of the diaphragm, while the Nakata index is the sum of the area of the LPA and RPA divided by the body surface [9, 10].

We compared fetal cardiac parameters according to the type of surgery—namely, single- or multistage surgery. Furthermore, we conducted a subgroup analysis in the single-stage surgery group according to the need for a secondary procedure or reoperation due to subsequent RVOT obstruction. We also compared fetal cardiac parameters between the two groups.

### Statistical analysis

Descriptive statistics were calculated, with continuous data presented as medians (ranges) and categorical variables presented as numbers (percentages). Parametric testing was used to compare data with normal distributions and comparisons were performed using the Student’s *t*-test. A receiver-operating characteristic (ROC) curve was used to obtain the cutoff value of each fetal cardiac parameter. The indicator of accurate prediction was the area under the curve (AUC). The cutoff value of each parameter was determined according to sensitivity and specificity. Data were assessed with the IBM SPSS Statistics version 26.0 software program (IBM Corp., Armonk, NY). *P*-values < 0.05 were considered to be statistically significant.

## Results

A total of 100 patients met the inclusion criteria for this study and the baseline characteristics are demonstrated in Table 1. Twelve neonates (12.0%) showed clinical symptoms related to RVOT obstruction immediately after birth, thus requiring medical support. All included patients underwent either single- (*N* = 90) or multistage surgery (*N* = 10). Among the 40 fetuses who underwent karyotyping, all showed normal results. Among the 31 fetuses undergoing multiplex ligation-dependent probe amplification for the detection of microdeletion syndromes, three were confirmed to have 22q11.2 deletion. 24 fetuses had right aortic arch and only one among CATCH 22 fetus had right aortic arch. Extracardiac anomalies were found in eight patients (8.0%)—these included renal diseases in three, gastrointestinal diseases in two, brain anomaly in one, spinal anomaly in one, and VACTERL association in one patient(s), respectively.

**Table 1:** Prenatal and postnatal characteristics of the fetuses with tetralogy of Fallot

Variable	Study population (*N* = 100)
Maternal age (years)	32 (23 ─ 43)
GA at diagnosis (weeks)	23.4 (19.6 ─ 27.6)
GA at follow-up (weeks)	32.5 (28.5 ─ 38.2)
GA at delivery (weeks)	39 (35.5 ─ 40.6)
Preterm birth	6 (6.0)
Birth weight (g)	3075 (1960 ─ 4182)
Apgar score at one minute (≥ 7)	90 (90)
Apgar score at five minutes (≥ 7)	100 (100)
Abnormal karyotype	0/40 (0)
22q11.2 deletion	3/31 (9.7)
Extracardiac anomalies	8 (8.0)
Neonatal death	1 (1.0)

Data are presented as medians (ranges) or numbers (percentages)

*GA* gestational age

Ninety patients underwent single-stage surgery at a median age of 5 months, while 10 underwent multistage surgery; in this latter group, the palliative shunt operation was conducted at a median age of 3 months and complete repair was performed at a median age of 10 months. Only one neonatal death occurred in an intrauterine growth-restricted newborn delivered at 38.3 weeks of gestation, who died as a result of sepsis after surgical correction.

### Comparison of parameters according to operation type

Fetal cardiac parameters measured in the second and third trimesters are described in Tables 2, where they are classified into the single- and multistage surgery groups. The PVA z-score, RPA z-score, and RPA/DAo ratio measured in the second trimester and the PVA z-score, RPA z-score, and PVA/AVA ratio measured in the third trimester were significantly lower in the multistage surgery group, while the PV-PSV as measured in both trimesters were significantly higher in the multistage surgery group.

**Table 2:** Comparison of parameters according to operation type at the second and third trimester

Parameter	Study population (*N* = 100)	Single-stage surgery (*N* = 90)	Multi-stage surgery (*N* = 10)	*P-*value
At the second trimester				
PVA z-score	−3.3 (−6.4 ─ 2.2)	−3.1 (−5.8 ─ 2.2)	−4.1 (− 6.4 ─ -1.8)	0.014
RPA z-score	−0.4 (− 3.1 ─ 1.8)	−0.3 (− 3.1 ─ 1.8)	−1.5 (− 2.0 ─ 0.3)	0.029
AVA z-score	0.5 (−1.8 ─ 3.3)	0.5 (− 1.8 ─ 3.3)	0.3 (− 1.7 ─ 3.2)	0.625
PV-PSV (m/s)	1.1 (0.7 ─ 1.6)	1.1 (0.7 ─ 1.6)	1.3 (1.2 ─ 1.6)	0.001
PVA/AVA	0.77 (0.41 ─ 1.39)	0.78 (0.50 ─ 1.39)	0.67 (0.41 ─ 1.00)	0.152
RPA/DAo	0.73 (0.45 ─ 1.12)	0.74 (0.45 ─ 1.12)	0.57 (0.52 ─ 0.81)	0.004
At the third trimester				
PVA z-score	−3.2 (−6.6 ─ -0.6)	− 3.0 (−5.4 ─ -0.6)	−5.0 (− 6.6 ─ -2.5)	< 0.001
RPA z-score	− 0.5 (− 2.6 ─ 1.2)	− 0.4 (− 2.6 ─ 1.2)	− 0.9 (− 2.2 ─ 0)	0.022
AVA z-score	1.0 (− 1.2 ─ 3.2)	1.0 (− 1.2 ─ 3.1)	0.9 (− 0.2 ─ 3.2)	0.732
PV-PSV (m/s)	1.2 (0.8 ─ 2.8)	1.2 (0.8 ─ 2.8)	1.5 (1.2 ─ 1.8)	0.002
PVA/AVA	0.76 (0.48 ─ 0.14)	0.77 (0.49 ─ 1.14)	0.60 (0.48 ─ 0.81)	< 0.001
RPA/DAo	0.72 (0.47 ─ 0.98)	0.72 (0.47 ─ 0.98)	0.66 (0.51 ─ 0.79)	0.058

Data are expressed as medians (ranges)

*PVA* pulmonary valve annulus, *RPA* right pulmonary artery, *AVA* aortic valve annulus, *PV-PSV* pulmonary valve peak systolic velocity, *DAo* descending aorta

**Fig. 2:**
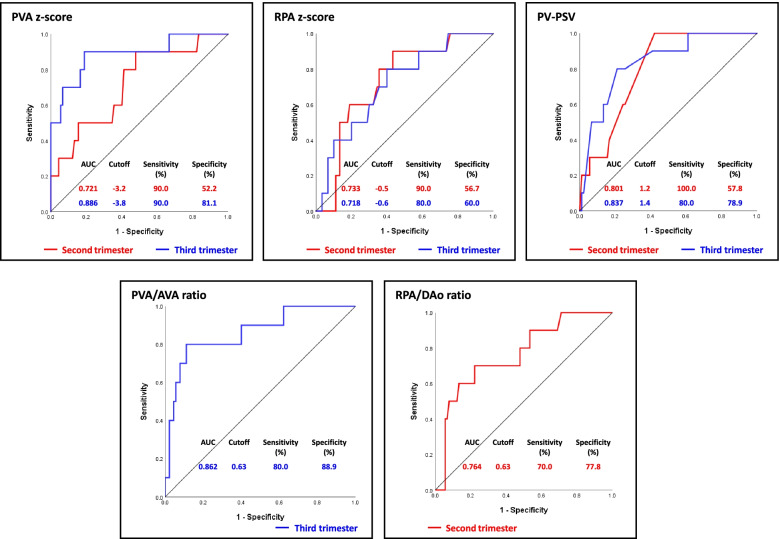
Receiver-operating characteristic curves for the fetal cardiac parameters and postnatal operation type. The presented cardiac ardiac parameters of note include the pulmonary valve annulus z-score (**A**), right pulmonary artery (RPA) z-score (**B**), pulmonary valve peak systolic velocity (**C**), pulmonary valve annulus/aortic valve annulus ratio (**D**), and RPA/descending aorta ratio (**E**). AUC, area under the receiver-operating characteristic curve

The ROC curves for the abovementioned meaningful parameters are presented in Fig. 2. The AUC values of all these parameters were greater than 0.700 and the PVA z-score measured in the third trimester achieved the highest AUC value of all (0.886). The optimal cutoff value of the PVA z-score measured in the third trimester was − 3.8 (sensitivity = 90.0% and specificity = 81.1%), while the optimal cutoff value of the RPA/DAo ratio measured in the second trimester was 0.63 (sensitivity = 70.0% and specificity = 77.8%).

### Subgroup analysis

The single-stage surgery group was further analyzed in accordance with the need for a secondary procedure or reoperation after the primary corrective surgery (Table 3). Of all patients in the group, 82 (91.1%) did not require a second procedure or operation because no complications occurred, while the remaining eight patients (8.9%) underwent balloon valvuloplasty or reoperation because of subsequent PA narrowing at the median age of 27 months. The PVA z-score and RPA z-score measured in both trimesters, PVA/AVA ratio in the third trimester, and RPA/DAo ratio in the second trimester were significantly different between the two groups. However, there was no significant difference in the PV-PSVs between the two groups when measured in either the second or third trimesters.

**Table 3:** Comparison of parameters according to the need for second procedure or reoperation

Parameter		Observation (*N* = 82)	Procedure or reoperation (*N* = 8)	*P-*value
PVA z-score	Second trimester	−3.0 (−5.8 ─ 2.2)	−4.1 (− 5.4 ─ -2.7)	0.049
	Third trimester	−3.0 (−5.3 ─ -0.6)	− 4.5 (− 5.4 ─ -2.7)	< 0.001
RPA z-score	Second trimester	−0.2 (− 3.1 ─ 1.8)	−1.1 (− 3.1 ─ 0.0)	0.031
	Third trimester	−0.4 (−2.6 ─ 1.2)	−1.1 (− 2.6 ─ 0.5)	0.004
PV-PSV (m/s)	Second trimester	1.1 (0.7 ─ 1.6)	1.3 (0.8 ─ 1.4)	0.342
	Third trimester	1.2 (0.8 ─ 2.8)	1.3 (0.8 ─ 1.5)	0.927
PVA/AVA RPA/DAo	Third trimester	0.78 (0.58 ─ 1.14)	0.67 (0.49 ─ 0.80)	0.003
	Second trimester	0.75 (0.48 ─ 1.12)	0.63 (0.45 ─ 0.81)	0.021

Data are expressed as medians (ranges)

*PVA* pulmonary valve annulus, *RPA* right pulmonary artery, *PV-PSV* pulmonary valve peak systolic velocity, *AVA* aortic valve annulus, *DAo* descending aorta

## Discussion

Our study demonstrates that several cardiac parameters could have predictive values for determining the postnatal operation type for TOF fetuses with the largest number of patients. The PVA z-score, RPA z-score, and PV-PSV in the second and third trimesters; PVA/AVA ratio in the third trimester; and RPA/DAo ratio in the second trimester are useful markers for the prediction of the postnatal operation type. Particularly, a PVA z-score of − 3.2 or less in the second trimester and − 3.8 or less in the third trimester; an RPA z-score of − 0.5 or less in the second trimester and − 0.6 or less in the third trimester; and a PV-PSV of 1.2 m/s or greater in the second trimester and 1.4 m/s or greater in the third trimester were associated with a high probability of requiring a palliative shunt operation followed by complete repair, whereas when these values were conversely above or below the mentioned cutoff, only single-stage surgery was required. Furthermore, we also suggest that a PVA/AVA ratio of 0.63 or less in the third trimester, and an RPA/DAo ratio—the new cardiac parameter suggested for the first time in the fetal period—of 0.63 or less in the second trimester may predict the need for a multistage surgery.

Our findings are consistent with those of previous studies that evaluated prenatal echocardiographic markers that may predict the outcomes of fetuses with TOF [11–13] [14] . Several studies reported that fetuses who required neonatal intervention had lower PVA z-scores and PVA/AVA ratios [11, 13] [14], and other research suggested that a PV-PSV of 144.5 cm/s or greater measured at 34 to 38 weeks of gestation supported the accurate prediction of early intervention and placement of transannular patches in 23 TOF fetuses [12]. However, in contrast with previous reports that did not report any significance of fetal RPA z-score in the prediction of postnatal outcomes of TOF fetuses [12, 13], our study found that the RPA z-score as measured in the second and third trimesters could also be a marker for predicting the postnatal operation type.

The RPA/DAo ratio is derived from the neonatal McGoon ratio, which is calculated using neonatal CT angiography and is used for quantifying the degree of PA hypoplasia [9] [10]. In the neonatal period, pediatric cardiologists commonly rely on this ratio to determine the best operation type for neonates with TOF. Hence, in this study, we created a new cardiac parameter by modifying the neonatal McGoon ratio, in which we only included the RPA rather than the sum of the LPA and RPA. RPA can be easily measured in the three-vessel view because it originates at the right angle and runs behind the ascending aorta, whereas the LPA runs the same course as the main PA, making it challenging to differentiate between the LPA and ductus arteriosus. Furthermore, as this study was retrospective in nature, many cases did not have LPA images available in their records.

During gestation, the affected structures in fetuses with TOF may change progressively [15]. For this reason, echocardiographic evaluations performed at later stages of pregnancy could contribute more so to elucidating key differences than those measured at earlier stages. However, our results did not fully correlate with this concept. This may be because accurate measurements are sometimes difficult to collect at advanced GA in that ultrasonographic scans may fail to provide accurate information because of poor image quality due to fetal positioning or ossification of the fetal chest. Furthermore, retrospective natrue of the study potentially affects this discordancy.

All infants underwent either single- or multistage surgery, with all but one of the patients included in this study surviving. As such, we confirmed an excellent prognosis associated with TOF. Our study also found that several infants who underwent single-stage surgery developed secondary pulmonary obstruction, requiring balloon valvuloplasty or surgical correction. A previous study reported that the PVA z-score could predict the need for reintervention after primary surgical correction [14]. In the present study, we found that not only the PVA z-score but also the RPA z-score, PVA/AVA ratio, and RPA/DAo ratio are valuable parameters in predicting the need for a second procedure or reoperation. Meanwhile, the PV-PSV as measured in both trimesters did not show statistical significance; however, because there were only eight patients who required further intervention, it might have been difficult to obtain significant findings. Future studies should be conducted involving larger numbers of patients.

This study has several strengths. First, it included the largest sample size to date compared to other similar studies at single center [12] [16] [17]. Second, the data were collected longitudinally from the second trimester to the third trimester and demonstrated that changes in cardiac parameters occurred with advancing GA. Through these longitudinally collected data, we compared various fetal cardiac parameters at each trimester and suggested the cutoff values of various parameters relevant at each trimester for predicting the type of surgical operation required. Third, we only included postnatally confirmed TOF, excluding pulmonary atresia with VSD and other diseases that cause outflow obstruction, to avoid heterogeneity of the study population. In addition, we suggested a new parameter (the modified McGoon ratio) for predicting the operation type for fetuses with TOF.

However, this study also has several limitations. First, it was a retrospective study conducted at a single center. In addition, the analysis was limited to only include patients with available images. Furthermore, only one investigator selected the most appropriate image and measured the cardiac parameters. As such, selection bias was possible.

## Conclusion

In conclusion, PVA z-score, RPA z-score, PV-PSV, PVA/AVA ratio, and RPA/DAo ratio demonstrate the potential to be useful parameters for predicting the necessary operation type for neonates with TOF. This information is clinically important because it can facilitate prenatal counselling and provide accurate data on the prognosis of neonates with TOF.

## Data Availability

The data and materials in this study are available from the corresponding author on request.

## Abbreviations

TOF: Tetralogy of Fallot
PVA: Pulmonary valve annulus
RPA: Right pulmonary artery
AVA: Aortic valve annulus
PV-PSV: Pulmonary valve-peak systolic velocity
DAo: Descending Aorta
VSD: Ventricular septal defect
PS: Pulmonary stenosis
RVOT: Right ventricular outflow tract
PA: Pulmonary artery
GA: Gestational age
ROC: Receiver-operating characteristic
AUC: Area under the curve
